# Identification of intraductal carcinoma of the prostate on tissue specimens using Raman micro-spectroscopy: A diagnostic accuracy case–control study with multicohort validation

**DOI:** 10.1371/journal.pmed.1003281

**Published:** 2020-08-14

**Authors:** Andrée-Anne Grosset, Frédérick Dallaire, Tien Nguyen, Mirela Birlea, Jahg Wong, François Daoust, Noémi Roy, André Kougioumoutzakis, Feryel Azzi, Kelly Aubertin, Samuel Kadoury, Mathieu Latour, Roula Albadine, Susan Prendeville, Paul Boutros, Michael Fraser, Rob G. Bristow, Theodorus van der Kwast, Michèle Orain, Hervé Brisson, Nazim Benzerdjeb, Hélène Hovington, Alain Bergeron, Yves Fradet, Bernard Têtu, Fred Saad, Frédéric Leblond, Dominique Trudel

**Affiliations:** 1 Centre de recherche du Centre hospitalier de l’Université de Montréal, Montreal, Quebec, Canada; 2 Institut du cancer de Montréal, Montreal, Quebec, Canada; 3 Department of Pathology and Cellular Biology, Université de Montréal, Montreal, Quebec, Canada; 4 Department of Computer Engineering and Software Engineering, Polytechnique Montréal, Montreal, Quebec, Canada; 5 Department of Engineering Physics, Polytechnique Montréal, Montreal, Quebec, Canada; 6 Department of Pathology, Centre hospitalier de l’Université de Montréal, Montreal, Quebec, Canada; 7 Laboratory Medicine Program, University Health Network, Toronto, Ontario, Canada; 8 Informatics & Biocomputing Program, Ontario Institute for Cancer Research, Toronto, Ontario, Canada; 9 Department of Human Genetics, University of California, Los Angeles, Los Angeles, California, United States of America; 10 Department of Urology, University of California, Los Angeles, Los Angeles, California, United States of America; 11 Institute for Precision Health, University of California, Los Angeles, Los Angeles, California, United States of America; 12 Jonsson Comprehensive Cancer Center, University of California, Los Angeles, Los Angeles, California, United States of America; 13 Princess Margaret Cancer Centre, University Health Network, Toronto, Ontario, Canada; 14 Oncology Division, Centre de recherche du Centre hospitalier universitaire de Québec–Université Laval, Quebec City, Quebec, Canada; 15 Centre de recherche sur le cancer, Université Laval, Quebec City, Quebec, Canada; 16 Department of Surgery, Université Laval, Quebec City, Quebec, Canada; Harvard Medical School, UNITED STATES

## Abstract

**Background:**

Prostate cancer (PC) is the most frequently diagnosed cancer in North American men. Pathologists are in critical need of accurate biomarkers to characterize PC, particularly to confirm the presence of intraductal carcinoma of the prostate (IDC-P), an aggressive histopathological variant for which therapeutic options are now available. Our aim was to identify IDC-P with Raman micro-spectroscopy (RμS) and machine learning technology following a protocol suitable for routine clinical histopathology laboratories.

**Methods and findings:**

We used RμS to differentiate IDC-P from PC, as well as PC and IDC-P from benign tissue on formalin-fixed paraffin-embedded first-line radical prostatectomy specimens (embedded in tissue microarrays [TMAs]) from 483 patients treated in 3 Canadian institutions between 1993 and 2013. The main measures were the presence or absence of IDC-P and of PC, regardless of the clinical outcomes. The median age at radical prostatectomy was 62 years. Most of the specimens from the first cohort (Centre hospitalier de l’Université de Montréal) were of Gleason score 3 + 3 = 6 (51%) while most of the specimens from the 2 other cohorts (University Health Network and Centre hospitalier universitaire de Québec–Université Laval) were of Gleason score 3 + 4 = 7 (51% and 52%, respectively). Most of the 483 patients were pT2 stage (44%–69%), and pT3a (22%–49%) was more frequent than pT3b (9%–12%). To investigate the prostate tissue of each patient, 2 consecutive sections of each TMA block were cut. The first section was transferred onto a glass slide to perform immunohistochemistry with H&E counterstaining for cell identification. The second section was placed on an aluminum slide, dewaxed, and then used to acquire an average of 7 Raman spectra per specimen (between 4 and 24 Raman spectra, 4 acquisitions/TMA core). Raman spectra of each cell type were then analyzed to retrieve tissue-specific molecular information and to generate classification models using machine learning technology. Models were trained and cross-validated using data from 1 institution. Accuracy, sensitivity, and specificity were 87% ± 5%, 86% ± 6%, and 89% ± 8%, respectively, to differentiate PC from benign tissue, and 95% ± 2%, 96% ± 4%, and 94% ± 2%, respectively, to differentiate IDC-P from PC. The trained models were then tested on Raman spectra from 2 independent institutions, reaching accuracies, sensitivities, and specificities of 84% and 86%, 84% and 87%, and 81% and 82%, respectively, to diagnose PC, and of 85% and 91%, 85% and 88%, and 86% and 93%, respectively, for the identification of IDC-P. IDC-P could further be differentiated from high-grade prostatic intraepithelial neoplasia (HGPIN), a pre-malignant intraductal proliferation that can be mistaken as IDC-P, with accuracies, sensitivities, and specificities > 95% in both training and testing cohorts. As we used stringent criteria to diagnose IDC-P, the main limitation of our study is the exclusion of borderline, difficult-to-classify lesions from our datasets.

**Conclusions:**

In this study, we developed classification models for the analysis of RμS data to differentiate IDC-P, PC, and benign tissue, including HGPIN. RμS could be a next-generation histopathological technique used to reinforce the identification of high-risk PC patients and lead to more precise diagnosis of IDC-P.

## Abstract 2

Andrée-Anne Grosset and colleagues use a novel spectroscopic approach to identify an aggressive histopathological variant of prostate cancer

## Author summary

**Why was this study done?:**

- Given its consistent association with prostate cancer (PC) recurrence, PC metastasis, and PC-specific death, the precise reporting of intraductal carcinoma of the prostate (IDC-P) is of the utmost importance.
- Pathologists nowadays rely mostly on morphology to differentiate intraductal lesions, with reported low interobserver agreement.
- Implementation of new methods in the clinical workflow would help reinforce the identification of high-risk PC patients and lead to more precise diagnosis of IDC-P.

**What did the researchers do and find?:**

- We used Raman micro-spectroscopy to identify the molecular composition of samples in the study of prostatic specimens.
- Spectral data retrieved from Raman micro-spectroscopy was analyzed using machine learning methods to generate predictive models based on biomolecular features to identify IDC-P, high-grade prostatic intraepithelial neoplasia (HGPIN), PC, and benign tissue.
- The tissue preparation protocol follows hospital standard operating procedures, facilitating implementation in clinical histopathology laboratories.

**What do these findings mean?:**

- This multicenter diagnostic accuracy case–control study showed Raman micro-spectroscopy combined with machine learning techniques could be used by pathologists to improve classification of intraductal lesions in PC.
- To substantiate the clinical implementation of Raman micro-spectroscopy, prospective validation studies including the full spectrum of intraductal lesions (i.e., from HGPIN to IDC-P including borderline lesions) will be necessary.

## Introduction

Prostate cancer (PC) is the most common cancer in North American men, and the second leading cause of death by cancer in men in the United States [1]. Diagnostics by pathologists involve visualizing hematoxylin and eosin (H&E)–stained 4-μm-thick tissue sections under the microscope, but there is a lack of reliable biomarkers to accurately characterize PC to ensure that precision medicine can benefit affected men [2]. Importantly, there are no clinically implemented biomarkers for the identification of intraductal carcinoma of the prostate (IDC-P), an aggressive variant of PC. In the vast majority of IDC-P cases, IDC-P occurs in combination with usual, invasive PC, and it is identified in approximately 20% of PC cases [3]. Given its consistent association with PC recurrence, PC metastasis, and PC-specific death, the precise reporting of IDC-P is of the utmost importance [3,4]. Molecular investigation of tumors or tumor regions with and without IDC-P have identified probabilistic differences in the frequency of different driver genes [5], in transcriptional and epigenomic profiles [6], in their microenvironment [7], in tumor evolutionary features [8], and in their visibility to multi-parametric magnetic resonance imaging [9]. However, once the intraductal nature of a prostatic lesion has been established, only morphological criteria can currently be used to diagnose IDC-P, resulting in reports of low interobserver concordance [10,11]. Importantly, IDC-P can be mistaken for other intraductal proliferations such as high-grade prostatic intraepithelial neoplasia (HGPIN), and vice versa [10,11]. These misinterpretations crucially affect the care of men with PC, as HGPIN and IDC-P are associated with opposite clinical significance, HGPIN being presumed to be a precursor of PC [11]. Biomarkers are available to solve this diagnostic pitfall, including phosphatase and tensin homolog (PTEN) loss of expression and ETS transcription factor ERG overexpression (both detected by immunohistochemistry [IHC]). However, these biomarkers have low sensitivity (60%–75%) and are thus not used frequently by genitourinary pathologists [12]. Reliable biomarkers of IDC-P with high sensitivity and specificity (>85%) would thus help reinforce the identification of such high-risk patients and lead to more appropriate patient management, ensuring the therapies are in line with IDC-P status [13–16].

Apart from standard molecular pathology techniques, tissue characterization methods have evolved with the use of optical microscopy, lately with a steep increase in data acquisition and data analysis capacities [17–21]. Among optical microscopy techniques, confocal Raman micro-spectroscopy (RμS) measures light scattering resulting from interactions with specific molecular bonds (among others, in proteins, lipids, DNA, and RNA), allowing for the global molecular characterization of a specimen [22]. The first RμS spectrum of normal prostate tissue was reported by Stone et al. in 2002, describing spectra acquired from snap-frozen tissues [23]. Subsequently, other groups reported the capacity to distinguish the different zones of the prostate as well as different prostatic cell lines, whether benign or malignant, with high sensitivity and specificity [24–26]. RμS has also been used on human tissues to successfully predict the occurrence of end-stage PC (i.e., castration-resistant PC) as well as to determine PC grade [27–30]. Reported results demonstrated the potential of RμS to identify PC; however, to our knowledge the technique has not been used to characterize subtypes of PC such as IDC-P.

Previous RμS studies were performed using snap-frozen samples, formalin-fixed paraffin-embedded (FFPE) tissues, or cytospin preparations of cell lines, deposited on expensive substrates such as CaF_2_, quartz, and gold-coated glass. Importantly, when performing a clinical diagnosis of PC, no tissue is available for snap freezing outside research purposes [31]. The previously reported RμS protocols for FFPE samples also involved tedious sample preparation, such as long dewaxing procedures or thick tissue sections. Those are key issues limiting clinical implementation of RμS.

As the currently available biomarkers of IDC-P are not sufficiently robust to be of clinical relevance and as RμS has been previously used to identify PC, but not IDC-P, we hypothesized that RμS could be used as a diagnostic biomarker of IDC-P. To investigate the central issue of identifying biomarkers of IDC-P, we developed a FFPE tissue slide preparation protocol that mirrors standard hospital procedures to facilitate clinical implementation of RμS for the characterization of PC. We then conducted a RμS study aimed at differentiating PC from benign prostate epithelium, as well as differentiating IDC-P from PC and benign prostatic epithelium, including HGPIN. This was achieved in FFPE radical prostatectomy specimens from 483 patients from 3 Canadian institutions.

## Methods

### Study overview

**Fig 1:**
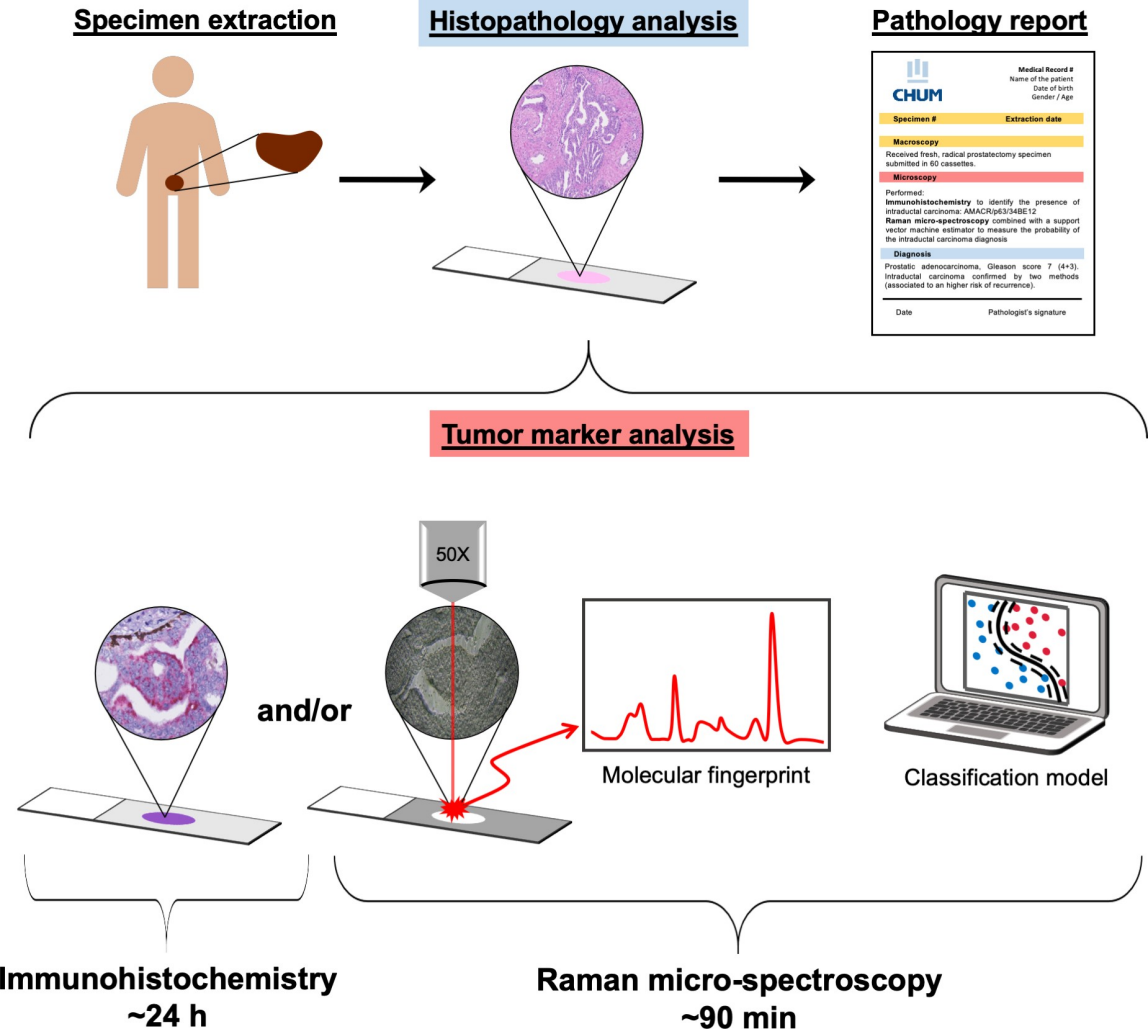
Integration of Raman micro-spectroscopy into the pathology workflow. Prostate cancer (PC) patients are often treated by first-line radical prostatectomy (whole specimen extraction). After surgery, the whole prostate is sent to the pathology department for routine analysis workflow: macroscopic visual examination, formalin fixation, paraffin embedding, microtome sectioning, and hematoxylin and eosin (H&E) staining. The pathologist examines H&E slides using a brightfield optical microscope (histopathology analysis) to determine the diagnosis before reporting. Ancillary analyses can increase the specificity of the diagnosis (e.g., identification of basal cells in benign prostatic tissues). The most frequently used complementary ancillary technique is immunohistochemistry (IHC), performed within approximately 24 hours. The cocktail of antibodies targeting α-methylacyl-CoA racemase (AMACR)/p63/34BE12 is applied to the prostate tissue to help identify benign glands and ducts, which are invaded by PC in the aggressive intraductal carcinoma of the prostate (IDC-P). However, no specific biomarker is available to identify IDC-P. Raman micro-spectroscopy (RμS) combined with a machine learning classification model can complement IHC in histopathology by providing a molecular fingerprint of the tissue that can predict the presence of IDC-P within 90 minutes. Technical aspects of the RμS workflow and graphical details associated with the machine learning workflow are shown in S1 and S2 Figs.

We conducted this study to present RμS as a promising, ancillary technique that could be integrated into the pathological workflow (Figs 1, S1 and S2). Tissue samples from 483 PC patients from 3 different institutions were studied: Centre hospitalier de l’Université de Montréal (CHUM), University Health Network (UHN), and Centre hospitalier universitaire de Québec–Université Laval (CHUQc-UL). FFPE tissue microarrays (TMAs) were used to allow high-throughput RμS acquisitions (S3 Fig). Two adjacent sections of each TMA block were cut. The first section was transferred onto a glass slide to perform IHC to detect AMACR/p63/34BE12 with H&E counterstaining [32]. The second section was placed on an aluminum slide with low Raman activity (Miro5011, Anomet, Brampton, ON, Canada). TMA sections on aluminum slides were dewaxed for 8 minutes according to the CHUM standard clinical dewaxing protocol. Briefly, slides were agitated for 1 minute in each bath: 2 xylene substitute baths (VWR, Radnor, PA, US), 3 100% ethanol baths (Alcools de Commerce, Boucherville, QC, Canada), and 3 distilled water baths. A vacuum dryer was used for 20 minutes to avoid residual water on the slides prior to RμS measurements, which were performed without any additional tissue processing.

IHC H&E-stained slides were digitized using a Nanozoomer Digital Pathology slide scanner (Hamamatsu, Bridgewater, NJ, US) before identification of lymphocytes, benign prostatic tissue, PC tissue, IDC-P, and HGPIN on the IHC-stained slide by 5 observers (AAG, TN, MB, JW, and FA, under the supervision of DT). A second pathologist (RA) confirmed the presence of IDC-P on targeted cores, ensuring all cases with morphological characteristics that failed diagnostic criteria were interpreted as negative for IDC-P [33].

Each acquisition using a Raman micro-spectrometer lasted for 50 seconds. All acquisitions were supervised by a research assistant specialized in RμS (MB). After all acquisitions were completed, support vector machine (SVM) classification models were trained with data from the CHUM cohort. In an SVM model, spectra are represented as points in a high-dimensional space where each dimension corresponds to a feature, i.e., a spectral wavelength shift associated with a distribution of Raman signal intensities. Support vectors are the basic entities or parameters computed by the algorithm to classify between different tissue classes. As support vectors are actual data points in the *N*-dimensional feature space, they have a geometrical interpretation with respect to the decision boundary, i.e., a multidimensional plane (a hyperplane with *N −* 1 dimensions), that separates the 2 targeted tissue classes. The SVM algorithm finds the optimal decision boundary, corresponding to the hyperplane that best separates the data into 2 classes within the feature space, by maximizing a loss function that depends on the geometrical distances of all data points to the decision boundary (S2 Fig). The SVM algorithm takes as input the following parameters, or hyperparameters: (i) the regularization parameter *C*, (ii) the kernel function (linear or Gaussian), and (iii) the kernel coefficient γ (see “Statistical analysis and tissue classification”). The SVM statistical model was then applied to 2 independent testing cohorts (UHN and CHUQc-UL). Our results determined the accuracy, sensitivity, and specificity of our statistical models to identify each cell and tissue type in PC patient samples. This study is reported as per diagnostic studies guidelines (STARD checklist; S1 Table).

For this study, a prospective analysis plan was constructed in 2015 as part of an internal grant covering RμS analysis of PC as well as other optical analysis of PC. Based on team discussions and external reviews, the analysis plan was reviewed in early 2019 to include a comparison between HGPIN and IDC-P. In 2020, following peer review comments, the revision of IDC-P by a second pathologist (RA) as well as confusion matrices analysis were also added.

### Human tissue samples

This multi-institutional retrospective study included a total of 483 PC patients and was approved by the CHUM ethics review board (15.107), after approval of the construction of the TMAs by local ethics review boards. All patients signed an informed consent allowing for the use of their prostate tissue samples in research. The TMAs from CHUM, UHN, and CHUQc-UL include patients treated by first-line radical prostatectomy and recruited from January 1, 1993, to December 31, 2013. FFPE PC tissues from surgery (radical prostatectomies) were used for the construction of TMAs, either by random selection of PC tissue (CHUM) or by targeted selection of representative grades (UHN and CHUQc-UL). All TMAs included benign tissues (tissue within normal, non-tumor range) from the radical prostatectomies performed to treat PC.

### Raman micro-spectroscopy

All Raman spectra were acquired using a Renishaw inVia confocal Raman microscope (Renishaw, Gloucestershire, UK) equipped with a 785-nm line focus laser. Each acquisition (4 acquisitions/TMA core) lasted 50 seconds (i.e., 5 accumulations of 10 seconds) with 150-mW laser output power using the 50× short working distance objective of the microscope (numerical aperture = 0.75). A rectangular area of 24 μm^2^ (8 μm × 3 μm, approximately corresponding to single-cell analysis) within cell-rich tissue, whether PC cells, IDC-P cells, benign epithelial cells, or lymphocytes, was targeted at each acquisition. Recognizable structures such as glandular lumens or the cobbly surface of cancer cell sheets were used to aim the laser on cells rather than extracellular matrix or areas without tissue such as glandular lumens. A grating of 1,200 lines/mm allowed the visualization of Raman shifts between 602 and 1,726 cm^−1^. Spatial registration of Raman spectra with the IHC H&E-stained slide ensured acquisitions were performed at the exact location of the cells from the tissues of interest. As aluminum slides are inevitably streaked, the final white-light tissue images on aluminum slides were processed with a filter in the frequency domain to avoid streak visualization. All Raman spectra files are available from the Dryad Digital Repository database [34].

### Statistical analysis and tissue classification

Aluminum background and intrinsic tissue fluorescence in the spectra were removed from the raw spectrum with the rolling ball algorithm [35]. As the resulting Raman spectra consisted of more than 1,000 spectral wavelengths shifts with a resolution of approximately 1.1 cm^−1^, a dimensional reduction procedure based on a linear SVM with L1 regularization was used prior to producing the classification models. This procedure allowed preselection of only those features (individual intensity values within a spectrum) that were most relevant in distinguishing tissue classes. The regularization method that was used, known as Lasso regression, assigns a weight to each feature within an optimization function (i.e., loss function) and gives a non-0 weight only to features that contribute significantly to establishing a decision boundary (S2 Fig).

Although SVM classifiers are linear classifiers, they can also be implemented using a nonlinear kernel function mapping the original data to another high-dimensional space, allowing improved classification performance by capturing more complex (i.e., nonlinear) attributes of the data. Here, the method used to produce the classification models from the preselected feature set was an SVM with a nonlinear Gaussian kernel. Prior to submitting the data to the classification algorithm [36], either for training or testing, each feature set underwent a standardization so that individual features had a mean of 0 and a unit variance.

Hyperparameters for the feature selection step and the subsequent classification model development were selected by performing a grid search. For the feature selection step, an SVM with a linear kernel was used with a regularization parameter *C* varying between 0.05 and 0.5, with larger values corresponding to more features being retained. For the development of the classification models (using only the preselected features), the regularization parameter *C* of the Gaussian SVM varied between 0.1 and 1,000, effectively acting as a penalty term for misclassified points. The kernel coefficient γ, which defines the variance of the Gaussian kernel, was varied between 10^−4^ and 10^−1^.

For each combination of hyperparameters (*C* and γ), the performance was assessed through 5-fold cross-validation. For this procedure, the training dataset was split into 5 nonoverlapping subsets. Each individual subset was used as a validation set while the other 4 were used to train a model, to assess the performance associated with a combination of hyperparameters. Cross-validation predictive performance was computed by averaging accuracy, sensitivity, and specificity across all folds, and the standard deviation was reported as modeling uncertainty. The model selected for testing was the one associated with hyperparameters yielding optimal classification. This final model was trained on the complete CHUM cohort and then tested on the UHN and CHUQc-UL cohorts. Training and testing performances were assessed through receiver operating characteristic (ROC) curves in which sensitivity and specificity were optimized by selecting the point with the minimal distance to the upper left corner (S5 Fig).

## Results

### RμS on TMAs

Three independent PC patient cohorts from Canadian hospitals were analyzed (Table 1). TMAs with core diameters ranging from 0.6 mm to 1.2 mm were assembled beforehand to ensure that all samples fit onto a small number of slides, to increase imaging throughput (S3 Fig) [37,38]. All Raman spectra were acquired using a confocal Raman microscope (inVia model, Renishaw, Gloucestershire, UK) equipped with a 785-nm line focus laser with an output power of 150 mW. Time for each acquisition was 50 seconds (5 accumulations of 10 seconds); the total time required for tissue processing and Raman spectra acquisition was <90 minutes for 1 patient. This was significantly shorter than other ancillary histopathology techniques, e.g., ~24 hours for IHC and several days for gene sequencing. Following a series of preprocessing steps including background removal, the Raman spectra were used to create classification models trained on the CHUM cohort and independently tested on the UHN and CHUQc-UL cohorts. Testing the model on data that were not used at any stage of the training phase ensures better clinical validity compared to internal validation strategies or the use of separate cohorts from a single institution for training and testing. Overall, 4 classification models were produced: The first identified lymphocyte clusters within prostate tissue, a classification that is reliably performed by pathologists; the second distinguished benign and malignant prostate epithelial cells to ensure recognition of cells from the same lineage; the third distinguished IDC-P from invasive carcinomas; and the fourth distinguished HGPIN from IDC-P for an accurate identification of the intraductal proliferation. Specifically, this protocol was developed to complement conventional pathology analyses in the identification of IDC-P (Figs 1, S1 and S2).

**Table 1:** Clinicopathological characteristics of patients from the 3 independent cohorts.

Characteristic	Institution		
	CHUM	UHN	CHUQc-UL
Number of patients	272	76	135
Median age in years at radical prostatectomy (IQR)	62 (58–66)	61 (57–66)	62 (59–67)
Median pre-operative PSA in μg/l (IQR)	7.4 (5.1–11.9)	6.9 (5.2–10.7)	6.6 (4.9–9.1)
Radical prostatectomy Gleason score, *n* (%)	265	67	133
≤3 + 3	139 (52)	14 (21)	10 (8)
3 + 4	74 (28)	34 (51)	69 (52)
4 + 3	22 (8)	14 (21)	42 (32)
≥4 + 4	30 (11)	5 (7)	12 (9)
Pathological tumor stage, *n* (%)	270	72	134
pT2	185 (69)	32 (44)	77 (57)
pT3a	60 (22)	32 (44)	41 (31)
pT3b	25 (9)	8 (11)	16 (12)
Presence of IDC-P among patients, *n* (%)	15 (6)	14 (18)	15 (11)
Raman micro-spectroscopy analysis			
Number of TMA cores per patient	1	1–3	1–6
Number of spectra per core	4	4	4
Number of spectra per patient	4	4–12	4–24

CHUM, Centre hospitalier de l’Université de Montréal; CHUQc-UL, Centre hospitalier universitaire de Québec–Université Laval; IDC-P, intraductal carcinoma of the prostate; IQR, interquartile range; PSA, prostate-specific antigen; TMA, tissue microarray; UHN, University Health Network.

### Identification of different cell types in PC tissue by RμS

Following the development of classification models for the detection of lymphocytes, 3 other classification models were developed to characterize PC and IDC-P: (i) benign versus cancer, (ii) IDC-P versus cancer, and (iii) HGPIN versus IDC-P. The potential of RμS to accurately differentiate cell types was quantified using a machine learning technique using as input (from the feature selection algorithm, see Methods) the Raman peaks contributing the most to the variability between different classes. As differentiation between lymphocytes and PC cells is straightforward in histopathology, we first tested the development of a RμS classification model to confirm the capacities of the system. This model could differentiate lymphocytes from PC cells with cross-validation accuracy, sensitivity, and specificity of 98%, 99%, and 98%, respectively (Methods; S2 Table). Classification performances were similar when testing the model on 2 independent cohorts. The peaks contributing the most to the classification models were assigned to vibrational modes and biochemical constituents (S4 Fig; S3 Table) [24–28,39,40].

**Fig 2:**
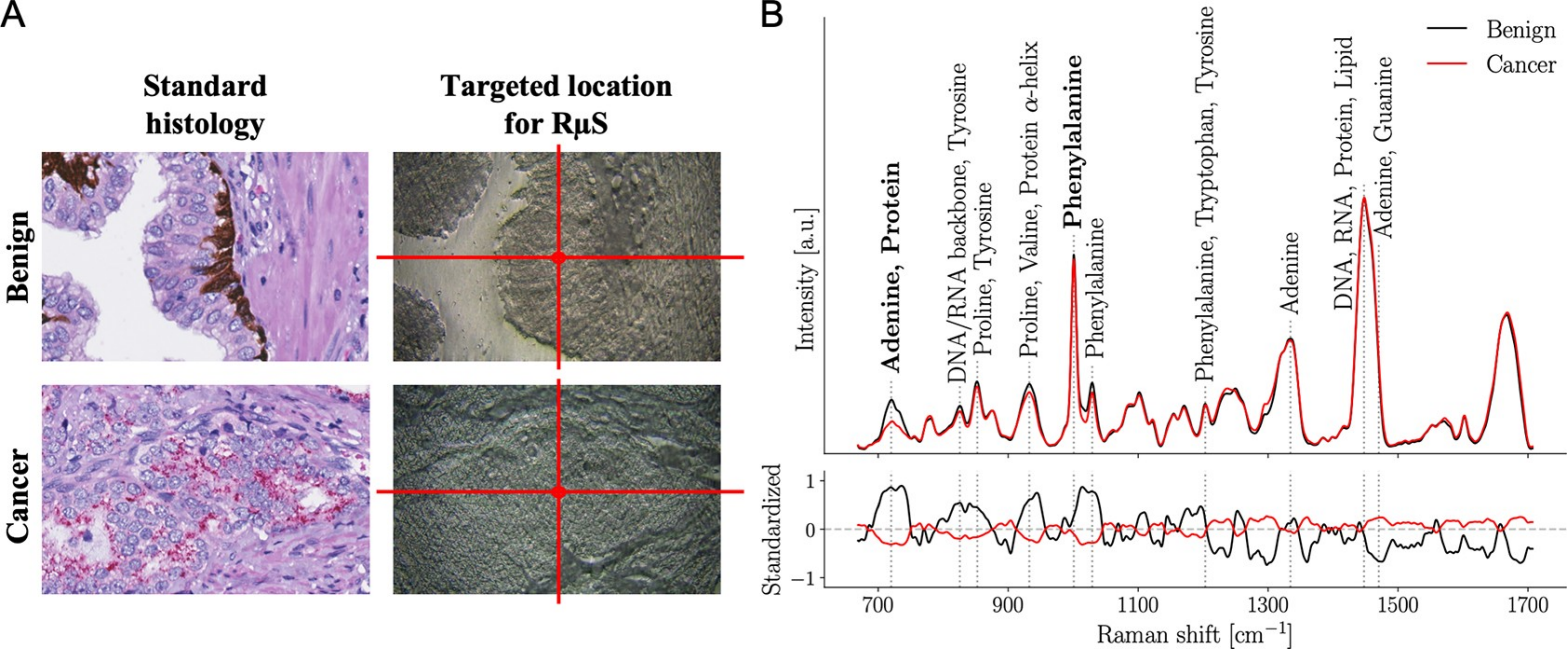
Prostate cancer diagnosis using Raman micro-spectroscopy. (A) Standard histology immunostaining for high molecular weight cytokeratins and p63 (basal cell markers in brown) and α-methylacyl-CoA racemase (cancer cell marker in red), followed by hematoxylin and eosin (H&E) counterstaining to identify benign prostatic glands and prostate cancer tissues. An adjacent 4-μm-thick tissue section slide was used to target a precise tissue point for Raman micro-spectroscopy (RμS) on unstained prostate tissue. (B) Average Raman spectra of benign prostatic glands (99 patients; 400 spectra) and prostate cancer (272 patients; 1,088 spectra) from the Centre hospitalier de l’Université de Montréal (CHUM) cohort. Raman peaks (i.e., biochemical constituents of tissue) that were dominant contributors to the classification are identified with dotted gray lines. Bottom frame shows the standardized Raman spectra, where each individual feature has 0 mean and unit variance. Spectra with their respective variance are shown in S6A Fig.

Machine learning/feature selection was then applied to distinguish benign prostate epithelial cells from PC cells. The diagnosis was determined by 1 pathologist (DT) on slides stained with antibodies targeting AMACR/p63/34BE12 to distinguish PC from benign glands [41] prior to RμS acquisitions on adjacent tissue sections (Fig 2A). The CHUM cohort, comprising 99 patients with benign prostatic tissue (400 spectra) and 272 patients with PC tissue (1,088 spectra), was used as the training set for the classification model. The Raman spectra for all CHUM patients were classified with an accuracy, sensitivity, and specificity of 87% ± 5%, 86% ± 6%, and 89% ± 8%, respectively (Table 2; S7A Fig). The model was then applied to the UHN cohort including 49 patients with benign prostatic tissue (196 spectra) and 76 patients with PC tissue (818 spectra). Performance on this testing cohort was comparable to that of the training dataset (CHUM) with an accuracy, sensitivity, and specificity of 84%, 84%, and 82%, respectively (S7B Fig). In the other testing cohort (CHUQc-UL), with 68 patients with benign prostatic tissue (272 spectra) and 135 patients with PC tissue (1,450 spectra), accuracy, sensitivity, and specificity were 86%, 87%, and 81%, respectively (S7C Fig).

**Table 2:** Classification performance when distinguishing benign prostate tissue, prostate cancer, IDC-P, and HGPIN in training and testing cohorts.

Performance measure	Classification performance, percent ± SD		
	Training CHUM	Testing	
		UHN	CHUQc-UL
**Benign/cancer**			
Accuracy	87 ± 5	84	86
Sensitivity	86 ± 6	84	87
Specificity	89 ± 8	82	81
AUC	87 ± 5	83	84
**IDC-P/cancer**			
Accuracy	95 ± 2	91	85
Sensitivity	96 ± 4	88	85
Specificity	94 ± 2	93	86
AUC	95 ± 3	90	85
**HGPIN/IDC-P**			
Accuracy	97.5 ± 1.4	97.8	98.3
Sensitivity	98.2 ± 1.5	95.5	96.4
Specificity	97.1 ± 1.4	100	100
AUC	97.6 ± 1.4	97.7	98.2

The classification was performed using an SVM with a Gaussian kernel coupled to a feature selection algorithm based on an SVM with a linear kernel and L1 regularization term. Confusion matrices for each classification are shown in S7 Fig for all training and testing sets.

AUC, area under the curve; CHUM, Centre hospitalier de l’Université de Montréal; CHUQc-UL, Centre hospitalier universitaire de Québec–Université Laval; HGPIN, high-grade prostatic intraepithelial neoplasia; IDC-P, intraductal carcinoma of the prostate; SVM, support vector machine; UHN, University Health Network.

We identified 32 important Raman spectral differences between benign and malignant prostate tissue, and those features were used to produce the machine learning classification models. From these, the 10 Raman peaks contributing the most to the classification of benign and cancer tissues were identified (Fig 2B; Table 3) [24–28,39,40]. The peaks at 1,450 cm^−1^ and 1,484 cm^−1^ were significantly increased in the average Raman spectrum of PC tissue compared to benign prostate tissue. Biochemical constituents assigned to these peaks were mostly from DNA and RNA, as well as from the backbone of proteins and from lipids. All other biochemical components of PC tissue identified by RμS were decreased compared to benign prostate tissue. More specifically, the nucleobase adenine from DNA and RNA, and the amino acids proline, tyrosine, valine, phenylalanine, and tryptophan were reduced in the average Raman spectrum of PC tissue.

**Table 3:** Most important features used for the classification of benign and malignant prostate tissue and their associated Raman peaks.

Feature (cm^−1^)	Peak center (cm^−1^)	Tissue type with increase	Main vibrational modes	Main molecules
720/733	719–726	Benign	Ring breathing mode, C-S	DNA/RNA (adenine), protein
828	827–828	Benign	O-P-O stretch, ring breathing	DNA/RNA backbone, protein (tyrosine)
841	853	Benign	C-C stretch, ring breathing	Protein (proline, tyrosine)
931	935–937	Benign	C-C stretch	Protein (proline, valine, α-helix)
1,012/1,013	1,000–1,003	Benign	Symmetric ring breathing	Protein (phenylalanine)
1,035	1,031–1,032	Benign	C-H stretch	Protein (phenylalanine)
1,200	1,206–1,207	Benign	C-C_6_H_5_ stretch	Protein (phenylalanine, tryptophan, tyrosine)
1,329	1,338	Benign	Unknown	DNA/RNA (adenine)
1,431	1,447–1,450	Cancer	CH_2_ deformation	DNA/RNA, protein, lipid
1,470	1,484	Cancer	Ring breathing mode	DNA/RNA (adenine, guanine)

The feature selection algorithm used was a linear SVM with L1 regularization. Tentative molecular assignment of prostate Raman peaks based on literature findings [24–28,39,40].

### RμS as a biomarker of IDC-P

**Fig 3:**
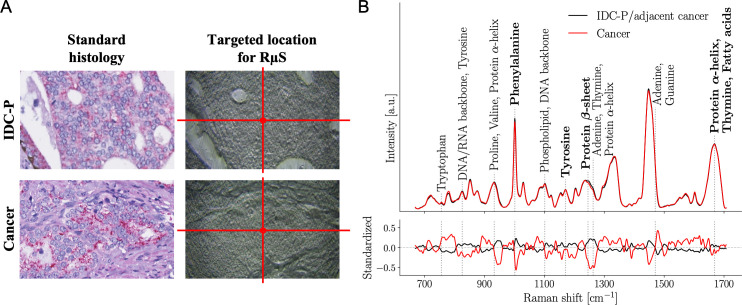
The presence of intraductal carcinoma of the prostate and adjacent usual invasive prostate cancer detected by Raman micro-spectroscopy. (A) Standard histology immunostaining for high molecular weight cytokeratins and p63 (basal cell markers in brown) and α-methylacyl-CoA racemase (cancer cell marker in red), followed by hematoxylin and eosin (H&E) counterstaining to identify intraductal carcinoma of the prostate (IDC-P) along with adjacent cancer and prostate cancer without IDC-P. An adjacent 4-μm tissue section on an aluminum slide was used to target a precise location for Raman micro-spectroscopy (RμS) on unstained prostate tissue. (B) Average Raman spectra of IDC-P with adjacent cancer (15 patients; 112 spectra) and prostate cancer (272 patients; 1,088 spectra) from the Centre hospitalier de l’Université de Montréal (CHUM) cohort. Raman peaks (i.e., biochemical constituents of tissue) that were dominant contributors to the classification are identified with dotted gray lines. Bottom frame shows the standardized Raman spectra, where each individual feature has 0 mean and unit variance. Spectra with their respective variance are shown in S6B Fig.

To train our statistical model, we used the cohort from CHUM. A total of 15 patients (17 cores) were identified with IDC-P (Fig 3A). When IDC-P was compared to adjacent invasive PC from the same core, RμS could not differentiate IDC-P from PC (in the UHN cohort, sensitivity was of 21%). Since several histopathological studies have reported that adjacent PC is similar to IDC-P [42–46], we combined the spectra of both regions of the prostate tissue, for a total of 112 spectra. We used the average Raman spectrum of each core with IDC-P and compared this to the average Raman spectrum of each PC core without IDC-P (272 patients, 1,088 spectra). The classification using machine learning (cross-validation) was performed, achieving an accuracy, sensitivity, and specificity of 95% ± 2%, 96% ± 4%, and 94% ± 2%, respectively (Table 2; S7D Fig). We then used our 2 other cohorts to test this classification model. From the UHN cohort, 14 patients were identified with IDC-P and adjacent PC on at least 1 TMA core. We acquired 139 Raman spectra from the 22 TMA cores from the 14 patients. For the classification, these spectra were compared to 767 PC spectra of TMA cores without IDC-P from 71 patients. Performances were comparable to cross-validation results from the training set, with an accuracy, sensitivity, and specificity of 91%, 88%, and 93%, respectively (S7E Fig). In the CHUQc-UL cohort, 9 patients (16 cores) with IDC-P were studied, and from these cores, we acquired 104 IDC-P spectra. PC from TMA cores without IDC-P from 93 patients was investigated, leading to 1,017 PC Raman spectra. The identification of IDC-P with the machine learning model using this cohort was performed with an accuracy, sensitivity, and specificity of 85%, 85%, and 86%, respectively (S7F Fig).

By comparing usual invasive PC to IDC-P, the feature selection algorithm retrieved 92 features from the training cohort. As when distinguishing benign versus cancer tissue, we identified the 10 most important Raman peaks that were used by machine learning (Fig 3B; Table 4) [24–28,39,40]. Specifically, the DNA and RNA backbone were increased in IDC-P compared to invasive PC. For proteins, α-helix and β-sheet secondary structures, specifically for the amide III peak, were more intense in the average Raman spectrum of IDC-P. Importantly, 3 features were associated with the amino acid tyrosine peak at 1,171 cm^−1^. Other amino acids (i.e., proline, valine, and phenylalanine) were identified mostly in IDC-P. We also observed a decrease in a few biochemical constituents in this aggressive variant of PC: the amino acid tryptophan (759 cm^−1^ peak), the nucleobase guanine, fatty acids, and the amide I peak from the protein α-helix (1,667 cm^−1^ peak).

**Table 4:** Most important features used for the classification of IDC-P and invasive prostate cancer tissue, and their associated Raman peaks.

Feature (cm^−1^)	Peak center (cm^−1^)	Tissue type with increase	Main vibrational modes	Main molecules
759	758–760	Cancer	Symmetric ring breathing	Protein (tryptophan)
834	827–831	IDC-P	O-P-O stretch, ring breathing	DNA/RNA backbone, protein (tyrosine)
952	935–937	IDC-P	C-C stretch	Protein (proline, valine, α-helix)
996	1,000–1,003	Cancer	Symmetric ring breathing	Protein (phenylalanine)
1,004	1,000–1,003	IDC-P	Symmetric ring breathing	Protein (phenylalanine)
1,108	1,090–1,100	IDC-P	O-P-O stretch	Lipid/phospholipid, DNA backbone
1,172/1,183/1,184	1,171	IDC-P	C-H bend	Protein (tyrosine)
1,250/1,251	1,242–1,250	IDC-P	Amide III	Protein (β-sheet)
1,266	1,263	IDC-P	Amide III	DNA/RNA (thymine, adenine), protein (α-helix)
1,477	1,484	Cancer	Ring breathing mode	DNA/RNA (adenine, guanine)
1,638/1,649	1,657–1,667	Cancer	C = O stretch, amide I	Protein (α-helix), lipid (fatty acid), DNA/RNA (thymine)

The feature selection algorithm used was a linear SVM with L1 regularization. Tentative molecular assignment of prostate Raman peaks based on literature findings [24–28,39,40].

IDC-P, intraductal carcinoma of the prostate.

**Fig 4:**
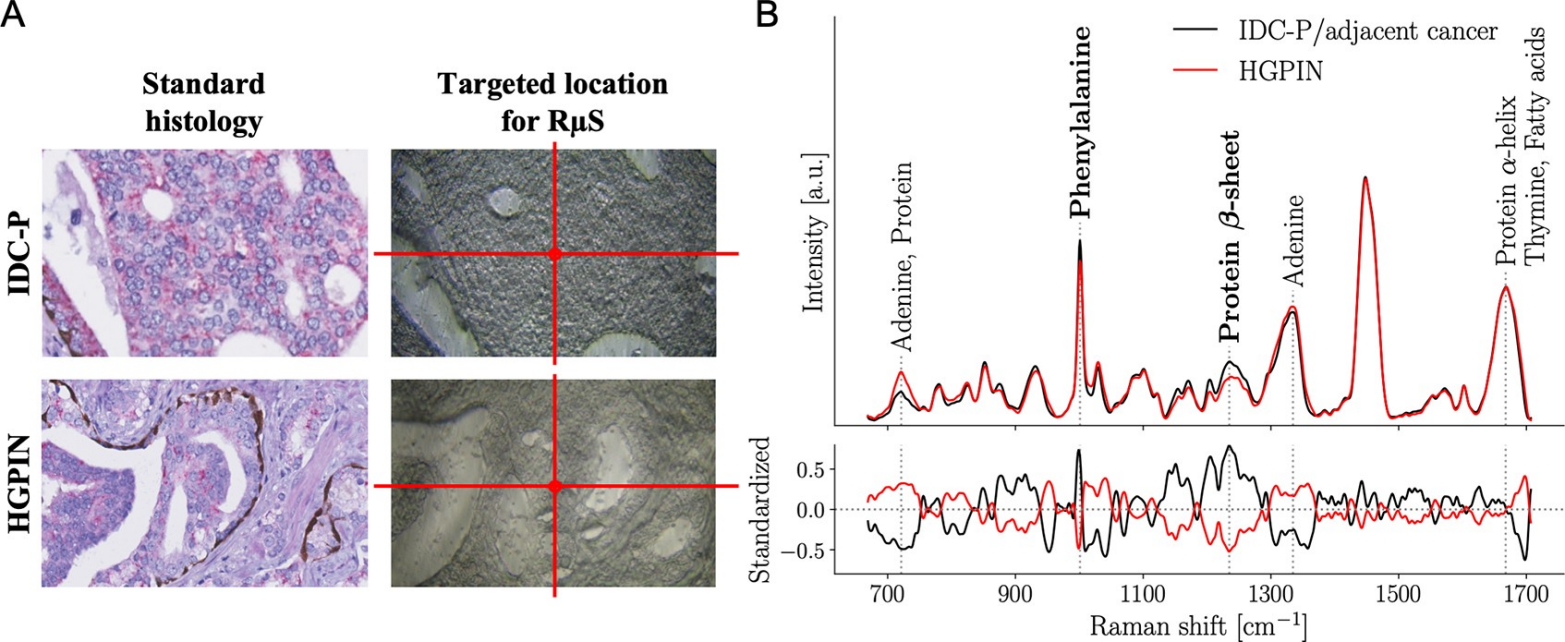
Raman micro-spectroscopy to accurately distinguish intraductal carcinoma of the prostate and high-grade prostatic intraepithelial neoplasia. (A) Standard histology immunostaining for high molecular weight cytokeratins and p63 (basal cell markers in brown) and α-methylacyl-CoA racemase (cancer cell marker in red), followed by hematoxylin and eosin (H&E) counterstaining to identify high-grade prostatic intraepithelial neoplasia (HGPIN) and intraductal carcinoma of the prostate (IDC-P) along with adjacent cancer. The images used to represent IDC-P are the same as those in Fig 3. An adjacent 4-μm tissue section on an aluminum slide was used to target a precise location for Raman micro-spectroscopy (RμS) on unstained prostate tissue. (B) Average Raman spectra of IDC-P with adjacent cancer (15 patients; 112 spectra) and HGPIN (64 patients; 170 spectra) from the Centre hospitalier de l’Université de Montréal (CHUM) cohort. Raman peaks (i.e., biochemical constituents of tissue) that were dominant contributors to the classification are identified with dotted gray lines. Bottom frame shows the standardized Raman spectra, where each individual feature has 0 mean and unit variance. Spectra with their respective variance are shown in S6C Fig.

We then tested the capacity of RμS to distinguish 2 intraductal proliferations, HGPIN and IDC-P (Fig 4A). The Raman spectra for all CHUM patients were classified (training, cross-validation) with an accuracy, sensitivity, and specificity of 97.5% ± 1.4%, 98.2% ± 1.5%, and 97.1% ± 1.4%, respectively (Table 2; S7G Fig). The model was then used on the UHN cohort, including 9 patients with HGPIN (23 spectra), and on the CHUQc-UL cohort, including 13 patients with HGPIN (30 spectra). Performance on testing cohorts was comparable to that of the training dataset (CHUM), with an accuracy of 97.8%–98.3%, a sensitivity of 95.5%–96.4%, and a specificity of 100% (S7H and S7I Fig).

For HGPIN and IDC-P, a total of 19 features were selected by the model. From these, we analyzed 5 Raman peaks that were the dominant contributors to the classification (Fig 4B; Table 5). The biochemical constituent predominantly found in HGPIN was adenine from DNA and RNA. Proteins were increased in IDC-P compared to HGPIN, more specifically the amino acid phenylalanine (1,003 cm^−1^ peak) and the β-sheet secondary structure (1,242 cm^−1^ peak).

**Table 5:** Most important features used for the classification of HGPIN and IDC-P, and their associated Raman peaks.

Feature (cm^−1^)	Peak center (cm^−1^)	Tissue type with increase	Main vibrational modes	Main molecules
720	725–726	HGPIN	C-S stretch, CH2 rocking	DNA/RNA (adenine), protein
1,000/1,008	1,000–1,003	IDC-P	Symmetric ring breathing	Protein (phenylalanine)
1,233/1,234	1,242–1,250	IDC-P	Amide III	Protein (β-sheet)
1,346	1,338	HGPIN	CH_3_CH_2_	DNA/RNA (adenine), collagen
1,696	1,657–1,667	HGPIN	C = O stretch, amide I	Protein (α-helix), lipid (fatty acid), DNA/RNA (thymine)

The feature selection algorithm used was a linear SVM with L1 regularization. Tentative molecular assignment of prostate Raman peaks based on literature findings [24–28,39,40].

IDC-P, intraductal carcinoma of the prostate; HGPIN, high-grade prostatic intraepithelial neoplasia.

## Discussion

Beyond distinguishing between PC and benign prostatic tissue with accuracy ≥84%, RμS indicated the presence of IDC-P within the diagnostic FFPE prostatic tissue with an accuracy of at least 85% throughout the 3 studied independent cohorts. Importantly, IDC-P could also be distinguished from HGPIN with accuracy >97%.

A precise diagnosis of IDC-P is a challenge for genitourinary pathologists [10,11], especially since no specific biomarker is clinically available to reliably identify this aggressive histological variant of PC. Indeed, once a lesion has been confirmed to be intraductal on a prostate biopsy, the most common biomarkers of IDC-P, ERG overexpression and PTEN loss, are used only by 25% of all genitourinary pathologists [12]. Here we showed RμS combined with machine learning technology could be used as a specific molecular biomarker of IDC-P, results that are in line with the described capacity to identify PC in FFPE and snap-frozen samples [24–26]. Interestingly, from the Raman peaks that were associated with IDC-P (Table 4), 2 were also associated with end-stage, castration-resistant PC by Wang et al. (1,171 cm^−1^ and 1,247 cm^−1^) [27]. As the association between IDC-P and castration-resistant PC is well established [15,47,48], we believe these results support the value of our classification models.

To ensure maximal clinical validity, we studied 3 nonoverlapping cohorts, composed of 76 to 272 PC patients, from different institutions to independently train and test our machine learning classification models. This study on RμS includes large cohorts of patients from different institutions, with stringent morphological and immunohistochemical classification of the lesions. Other groups have conducted PC RμS studies on only 1 patient or on single-center cohorts composed of a maximum of 50 patients [23–30,49]. Studying a large group of men with PC from different institutions paves the way to the use of Raman spectra as a biomarker of IDC-P.

In addition to showing the capacity to detect a diagnostic signature for a histological variant of PC, our RμS protocol is fully compatible with the standard clinical histopathological workflow. First, in contrast to other published protocols using fresh or frozen tissues, our analyses were performed using FFPE tissues. Because PC is most often visually undetectable and impalpable, the entirety of prostate specimens examined in the specific context of PC diagnosis are FFPE in toto, i.e., no tissue is available for snap freezing outside research purposes [31]. Importantly, blindly harvesting tissue to eventually perform RμS on snap-frozen tissue—a method that is adopted for other organ systems instead of FFPE-based evaluations—could lead to analyzing a tissue devoid of PC or, even worse, underestimating disease severity if significant portions of the tumor are unavailable for routine H&E evaluation.

Second, we developed our protocol to enable smooth implementation into a clinical laboratory setting without disruption of routine service. Cut at the same thickness as standard tissue sections (4 μm) and dewaxed using our regular routine dewaxing protocol, RμS tissue sections can be treated using the same apparatus at the same settings as the vast majority of the tissue sections processed in a histopathology laboratory. The aluminum slides we used were of the same size as standard microscope glass slides and compatible with all chemicals used to dewax/prepare the sample for RμS and are in addition inexpensive. Tissue sections do not need further labeling before RμS acquisitions. Classification models can also be applied without technical engineering assistance with only basic training in the use of a RμS microscope. The cost of a Raman confocal microscope is in the same range as the cost of an automated immunostainer to perform IHC, and a Raman confocal microscope is also smaller than an automated autostainer. Therefore, a laboratory setup in which some slides are analyzed by standard molecular pathology techniques (e.g., IHC) while others are sent for RμS evaluation can be envisioned with minimal disruption of the standard clinicopathological workflow.

As our results involve large-scale validation and the use of a clinically implementable slide preparation protocol, the inclusion of lesions that fall short of the diagnostic criteria of IDC-P would have improved the clinical significance of our results. Indeed, these difficult-to-classify lesions are the lesions for which pathologists are expected to use RμS. However, as IDC-P was present in 6% to 18% of the investigated patients, as expected by the small diameter of the TMA cores and the limited size of IDC-P within PC [50], the number of borderline, difficult-to-classify lesions in our TMA set was insufficient for proper classification. Importantly, the RμS algorithms we designed provide a quantitative evaluation of the probability of a diagnosis (e.g., IDC-P, 75% probability), therefore decreasing the impact of this limitation.

Before clinical implementation, in which RμS could be used similarly to IHC (Figs 1, S1 and S2), next steps for research include, among others, validation in cohorts using material entirely processed in each center (from tissue fixation to slide preparation to RμS acquisitions) to ensure reproducibility of all the steps of the protocol regardless of the laboratory. Importantly, a thorough validation of the use of RμS in different disease classifications and organ types [51] will also facilitate the clinical implementation of RμS, by maximizing the use of the Raman microscopes. Moreover, other RμS modalities such as surface-enhanced Raman spectroscopy (SERS) or coherent anti-Stokes Raman spectroscopy (CARS) could be tested to improve the acquisition speed (allowing one to analyze larger portions of the specimens) and/or to modify the substrate on which RμS is performed to allow the use of glass slides. Altogether, RμS is a promising tool for histopathological ancillary studies, but further large-scale, multicenter studies are needed before actual clinical implementation.

The extent of similarity between IDC-P and immediately adjacent PC will also have to be investigated. Indeed, when IDC-P was present in a core, the Raman spectra from PC and IDC-P on the core were indistinguishable, despite their different localization with respect to prostatic ducts (inside for IDC-P, outside for PC). We thus combined the spectra from IDC-P and from the adjacent invasive PC tissue from the same core. As IDC-P and immediately adjacent PC have been shown to have similar expression of biomarkers such as ERG and PTEN [42–46], this similarity is not unexpected. However, beyond the scientific phylogeny questions raised by these similarities, from a technical standpoint, it will be of tremendous importance to characterize the “Raman-identical” zone around IDC-P. Among other factors, the size of this zone is likely to define the needed precision when evaluating an intraductal lesion.

Overall, we provided a large study of the use of RμS to detect PC and IDC-P in 3 independent cohorts of men with PC. Our results are not only in line with the current literature associating the important Raman features of IDC-P with the development of castration-resistant PC, but they also provide solid evidence to pursue the clinical implementation of RμS as an ancillary technique to refine the diagnosis of PC. In perspective, a prospective study on fully annotated specimens, including difficult-to-classify lesions, will ensure the transition from the testing of research TMAs to clinical workflow.

## Supporting information

S1 Fig RμS workflow. The localization of the tissue for the RμS acquisition is done by looking simultaneously at the digitalized-stained-annotated TMAs and the sample viewer from Renishaw WiRE software. Adjusting the position of the laser on the sample is done by moving the stage of the microscope; the position of the laser is seen on the sample view of the WiRE software. Via this adjustment, the laser is correctly positioned on the cell(s) of the tissue to be probed.
(TIF)

S2 Fig Machine learning workflow. The workflow of the classification is read from top to bottom. In our analysis, features are spectral wavelengths (e.g., 1,004 cm^−1^, 1,477 cm^−1^) with a corresponding value (Raman intensity) different for each Raman spectrum. The feature selection algorithm is a linear SVM with a L1 regularization. As it assigns a weight to each feature, only features contributing to the decision boundary are assigned a non-zero weight. The classification algorithm is an SVM with a Gaussian kernel that maps the original feature set to a different high-dimensional space in which data are linearly separable.
(TIF)

S3 Fig Prostate cancer tissue microarray. A representative standard histology immunostaining of a TMA for high molecular weight cytokeratins and p63 (basal cell markers in brown) and α-methylacyl-CoA racemase (cancer cell marker in red), followed by H&E counterstaining to identify low-grade PC (contoured in green), high-grade PC (contoured in red), IDC-P (contoured in yellow, as well as other intraductal atypical lesion), lymphocytes (contoured in white), and a focus of perineural invasion (contoured in black). Cores with uniform morphology were investigated but not contoured. Black dots indicate RμS measurement locations.
(TIF)

S4 Fig Identification of lymphocyte clusters in PC tissue by RμS. (A) Standard histology immunostaining for high molecular weight cytokeratins and p63 (basal cell markers in brown) and α-methylacyl-CoA racemase (cancer cell marker in red), followed by H&E counterstaining to identify lymphocytes and PC tissues. An adjacent 4-μm tissue section on aluminum Miro5011 slide was used to target a precise tissue point for RμS on unstained prostate tissue (image modified to enhance tissue visualization). (B) Average Raman spectra of lymphocytes (40 patients; 168 spectra) and PC (272 patients; 1,088 spectra) from the CHUM cohort. Raman peaks (i.e., biochemical constituents of the tissue) that were dominant contributors to the classification are identified through a linear SVM with L1 regularization and shown with dotted gray lines. Biochemical constituents are expressed in bold when multiple features are associated with a single Raman peak. Bottom frame shows the standardized Raman spectra, where each individual feature has 0 mean and unit variance.
(TIF)

S5 Fig Receiver operating characteristic curves. Receiver operating characteristic (ROC) curves for benign prostatic glands and PC (A), IDC-P with adjacent cancer and PC (B), and IDC-P with adjacent cancer and HGPIN (C). CHUM training set is indicated with a solid line, whereas UHN and CHUQc-UL testing sets are denoted with a dashed line and a dotted line, respectively. Red dots correspond to the point that is the closest to the upper left corner—associated with maximum sensitivity and specificity—and represent values that optimize sensitivity and specificity for each set; threshold values associated to each figure are 0.75 (A), 0.25 (B), and 0.33 (C).
(TIF)

S6 Fig Average spectra and respective variance. Average Raman spectra of benign prostatic glands and PC (A), IDC-P with adjacent cancer and PC (B), and IDC-P with adjacent cancer and HGPIN (C) from the CHUM cohort. Average spectra are shown (bold) with their associated variance (shaded area). Raman peaks (i.e., biochemical constituents of the tissue) that were dominant contributors to the classification were identified through a linear SVM with L1 regularization and are shown with dotted gray lines.
(TIF)

S7 Fig Confusion matrices. Confusion matrices associated with models differentiating between benign tissue, PC, IDC-P, and HGPIN in training and testing cohorts. In each panel (A–I), columns represent the predicted numbers for a given class while rows represent the numbers belonging to their true class (pathological labels). These numbers allow extraction of true positive, true negative, false positive, and false negative rates for each model in both training and testing sets. Numbers in each cell represent the number of cores, except for IDC-P in (D–G) and HGPIN in (G), which correspond to the total number of spectra.
(TIF)

S1 Table The STARD checklist. (DOCX)

S2 Table Classification performance when distinguishing lymphocyte clusters and PC in training and testing cohorts. (DOCX)

S3 Table Most important features used for the classification of lymphocytes and cancer within prostate tissue and their associated Raman peaks. (DOCX)

## Abbreviations

CHUM: Centre hospitalier de l’Université de Montréal
CHUQc-UL: Centre hospitalier universitaire de Québec–Université Laval
FFPE: formalin-fixed paraffin-embedded
H&E: hematoxylin and eosin
HGPIN: high-grade prostatic intraepithelial neoplasia
IDC-P: intraductal carcinoma of the prostate
IHC: immunohistochemistry
PC: prostate cancer
RμS: Raman micro-spectroscopy
SVM: support vector machine
TMA: tissue microarray
UHN: University Health Network
