# Supplementary figures and images for: Identification of intraductal carcinoma of the prostate on tissue specimens using Raman micro-spectroscopy: A diagnostic accuracy case–control study with multicohort validation
Source: PLoS Med. 2020 Aug 14;17(8):e1003281. doi: 10.1371/journal.pmed.1003281 (PMC7428053; doi:10.1371/journal.pmed.1003281)

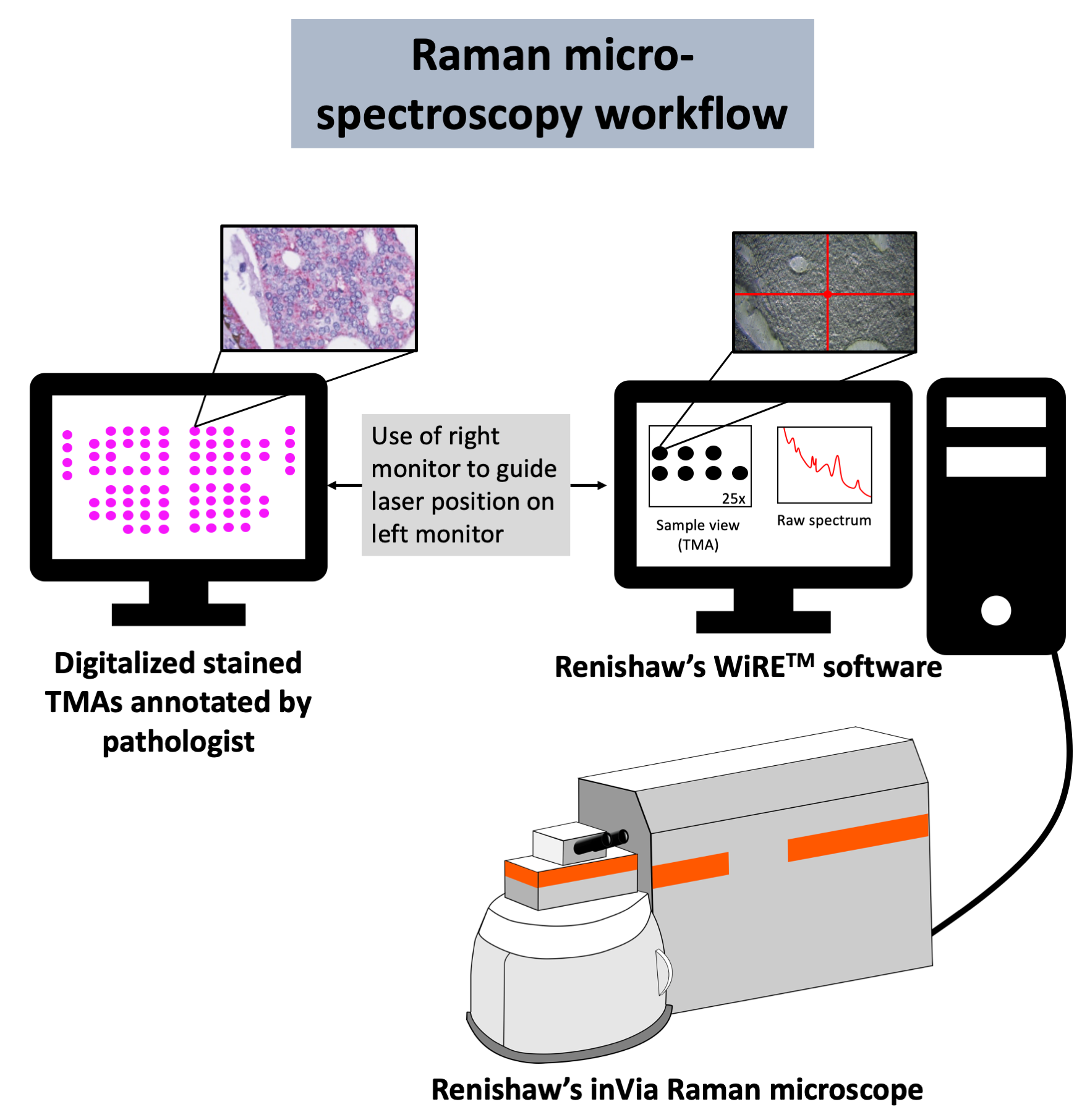

Supplement: S1 Fig — The localization of the tissue for the RμS acquisition is done by looking simultaneously at the digitalized-stained-annotated TMAs and the sample viewer from Renishaw WiRE software. Adjusting the position of the laser on the sample is done by moving the stage of the microscope; the position of the laser is seen on the sample view of the WiRE software. Via this adjustment, the laser is correctly positioned on the cell(s) of the tissue to be probed. (TIF) [file pmed.1003281.s001.tif]

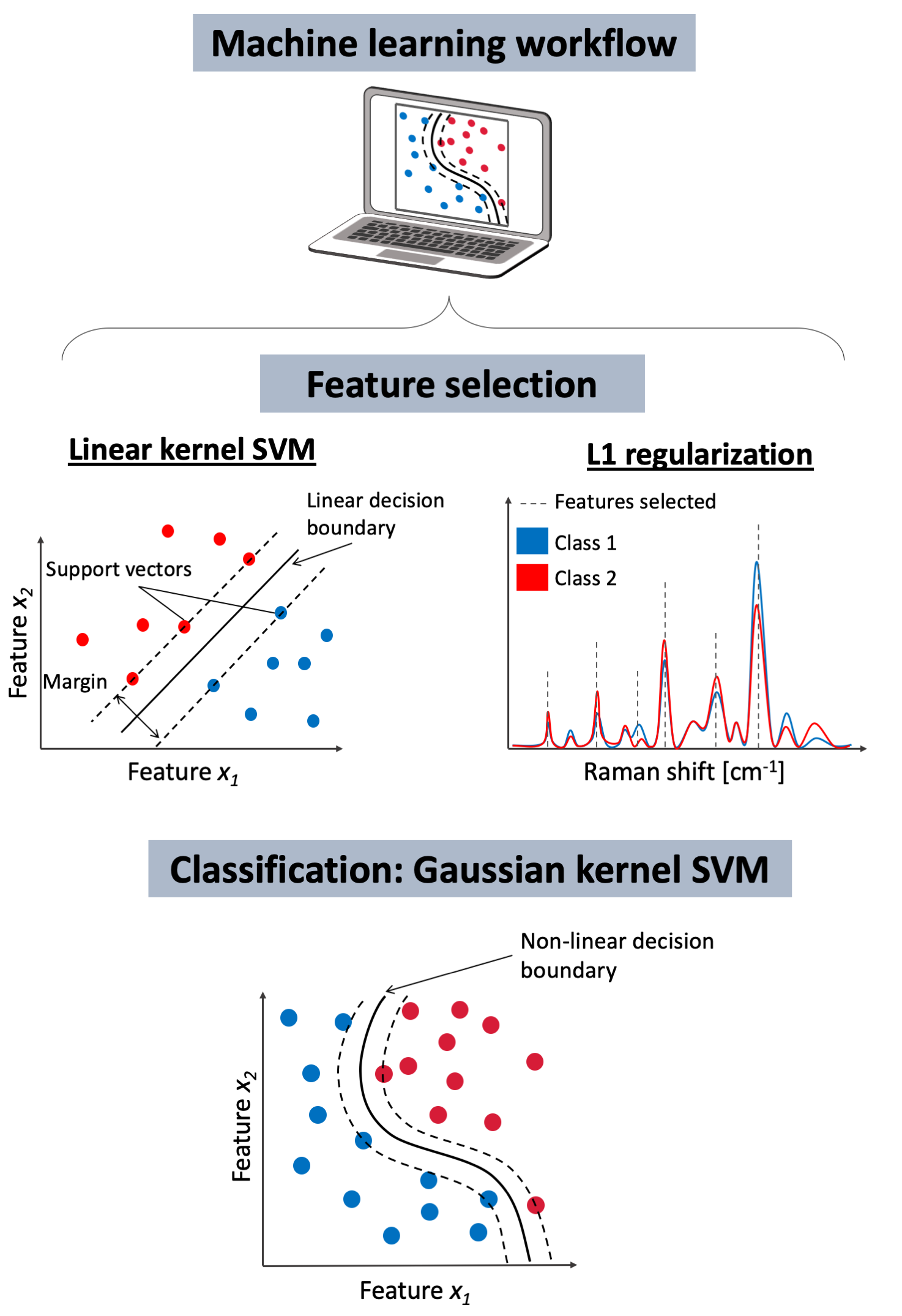

Supplement: S2 Fig — The workflow of the classification is read from top to bottom. In our analysis, features are spectral wavelengths (e.g., 1,004 cm−1, 1,477 cm−1) with a corresponding value (Raman intensity) different for each Raman spectrum. The feature selection algorithm is a linear SVM with a L1 regularization. As it assigns a weight to each feature, only features contributing to the decision boundary are assigned a non-zero weight. The classification algorithm is an SVM with a Gaussian kernel that maps the original feature set to a different high-dimensional space in which data are linearly separable. (TIF) [file pmed.1003281.s002.tif]

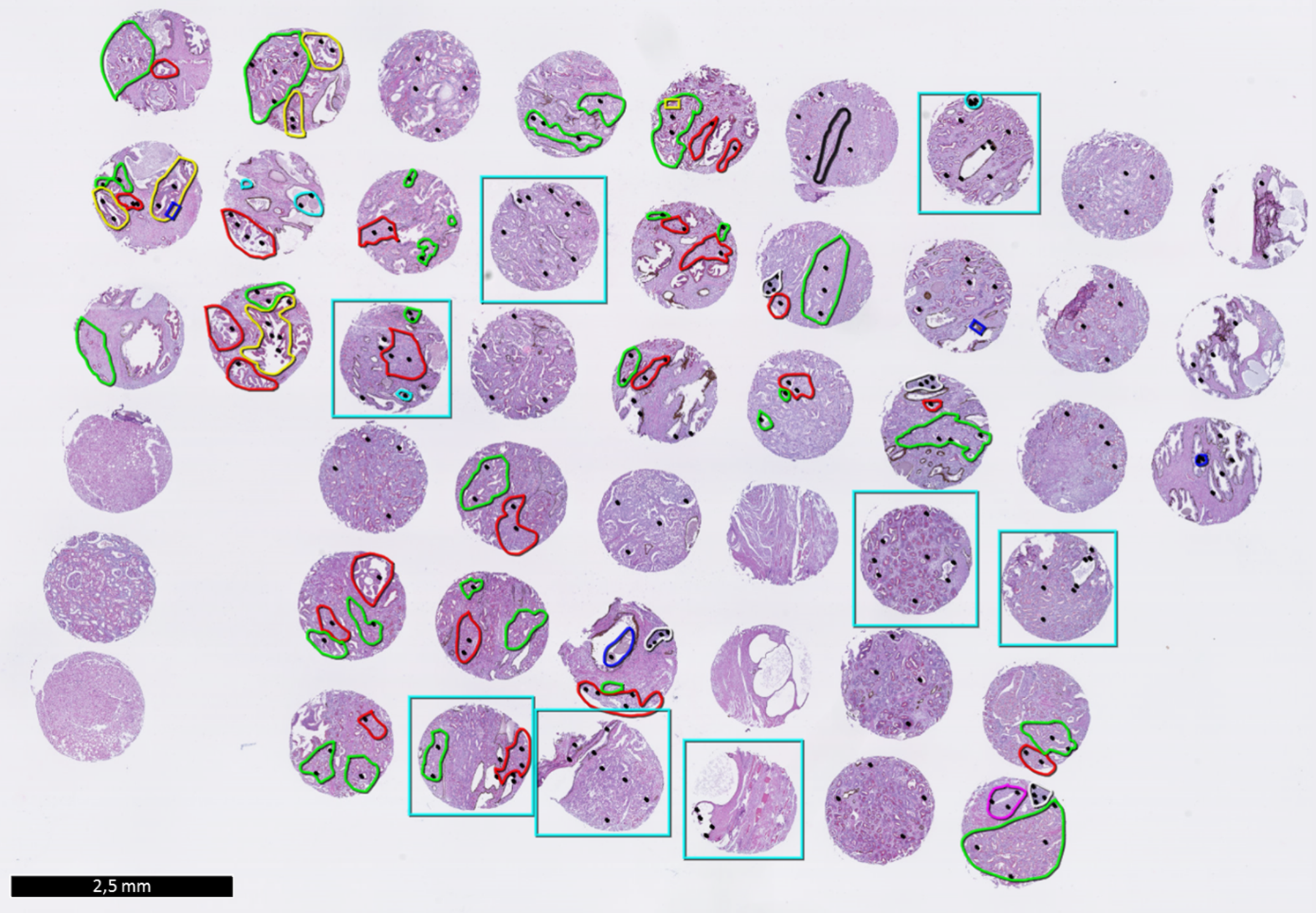

Supplement: S3 Fig — A representative standard histology immunostaining of a TMA for high molecular weight cytokeratins and p63 (basal cell markers in brown) and α-methylacyl-CoA racemase (cancer cell marker in red), followed by H&E counterstaining to identify low-grade PC (contoured in green), high-grade PC (contoured in red), IDC-P (contoured in yellow, as well as other intraductal atypical lesion), lymphocytes (contoured in white), and a focus of perineural invasion (contoured in black). Cores with uniform morphology were investigated but not contoured. Black dots indicate RμS measurement locations. (TIF) [file pmed.1003281.s003.tif]

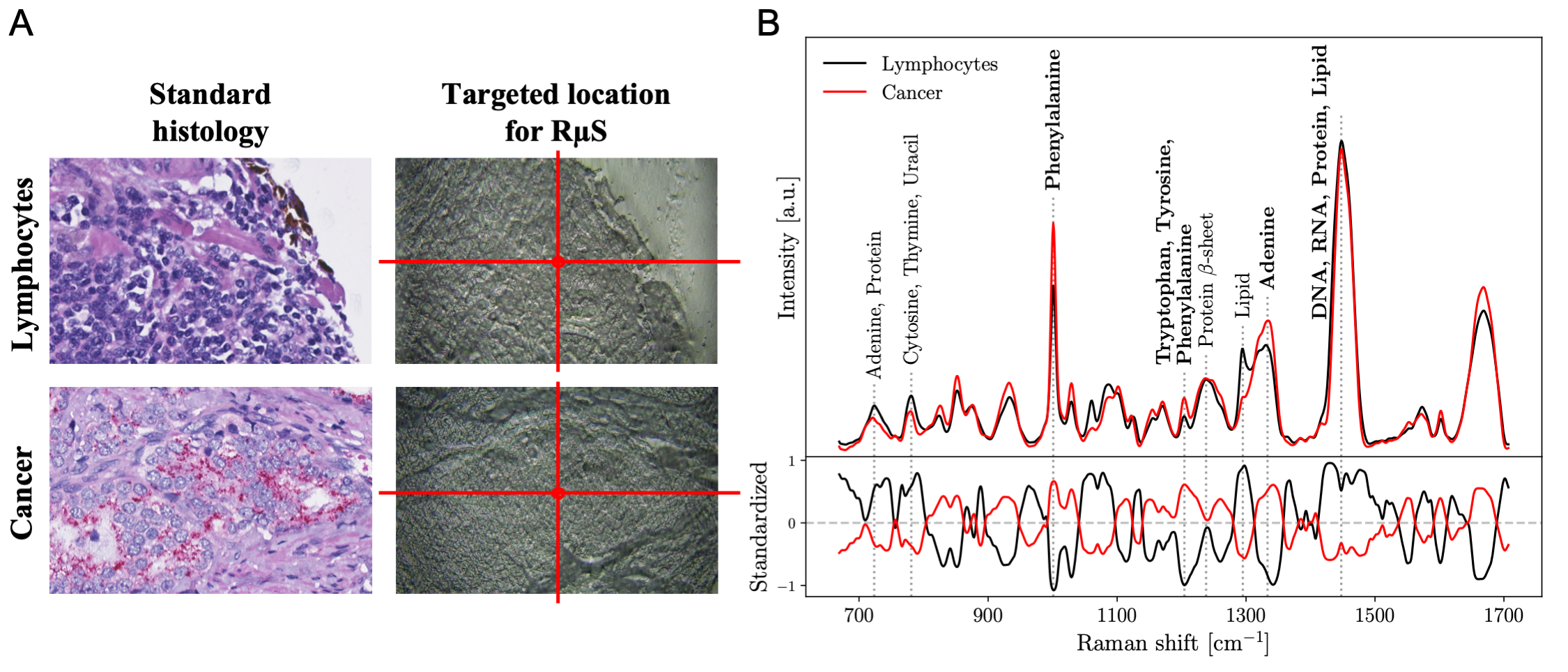

Supplement: S4 Fig — (A) Standard histology immunostaining for high molecular weight cytokeratins and p63 (basal cell markers in brown) and α-methylacyl-CoA racemase (cancer cell marker in red), followed by H&E counterstaining to identify lymphocytes and PC tissues. An adjacent 4-μm tissue section on aluminum Miro5011 slide was used to target a precise tissue point for RμS on unstained prostate tissue (image modified to enhance tissue visualization). (B) Average Raman spectra of lymphocytes (40 patients; 168 spectra) and PC (272 patients; 1,088 spectra) from the CHUM cohort. Raman peaks (i.e., biochemical constituents of the tissue) that were dominant contributors to the classification are identified through a linear SVM with L1 regularization and shown with dotted gray lines. Biochemical constituents are expressed in bold when multiple features are associated with a single Raman peak. Bottom frame shows the standardized Raman spectra, where each individual feature has 0 mean and unit variance. (TIF) [file pmed.1003281.s004.tif]

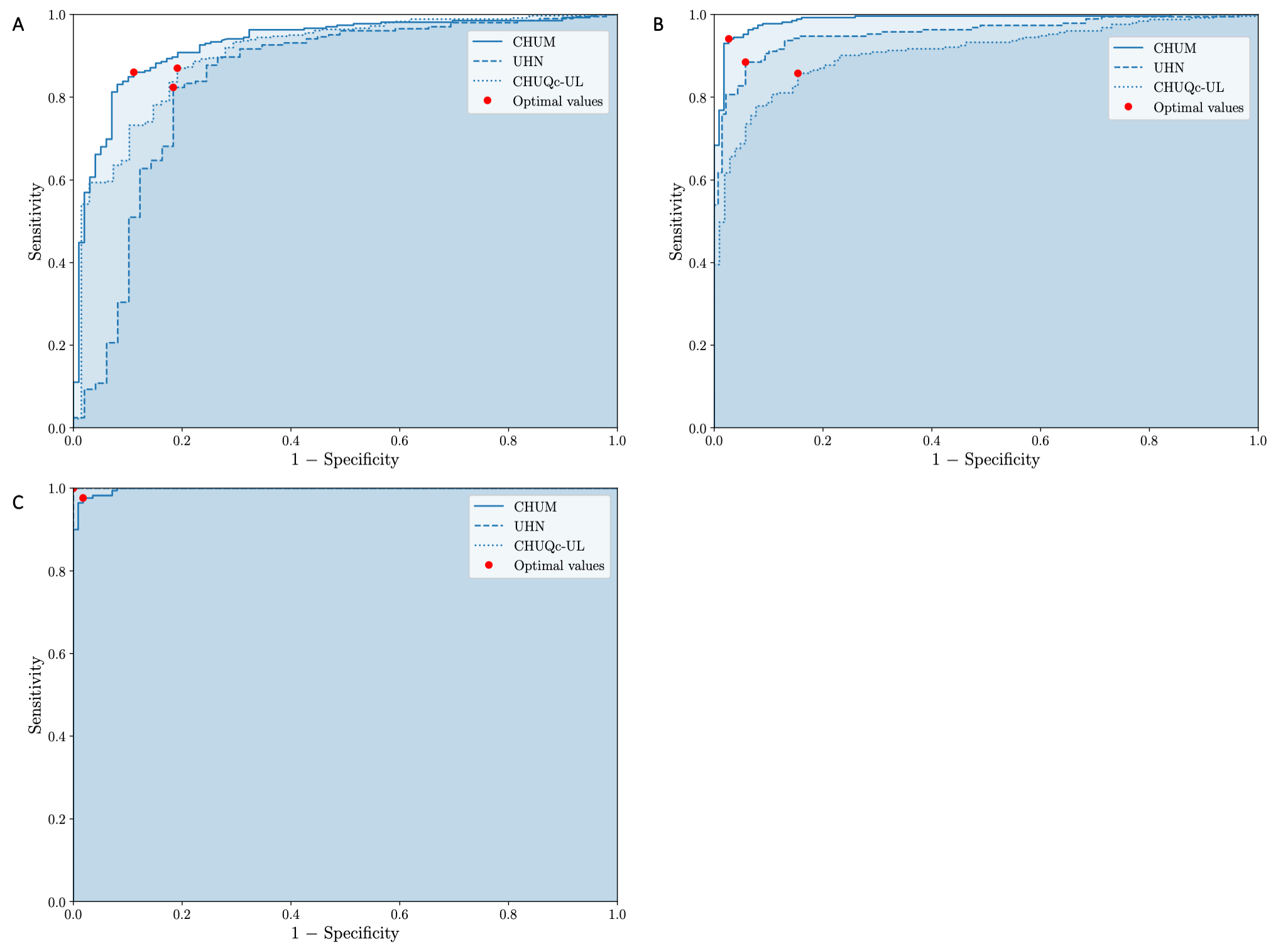

Supplement: S5 Fig — Receiver operating characteristic (ROC) curves for benign prostatic glands and PC (A), IDC-P with adjacent cancer and PC (B), and IDC-P with adjacent cancer and HGPIN (C). CHUM training set is indicated with a solid line, whereas UHN and CHUQc-UL testing sets are denoted with a dashed line and a dotted line, respectively. Red dots correspond to the point that is the closest to the upper left corner—associated with maximum sensitivity and specificity—and represent values that optimize sensitivity and specificity for each set; threshold values associated to each figure are 0.75 (A), 0.25 (B), and 0.33 (C). (TIF) [file pmed.1003281.s005.tif]

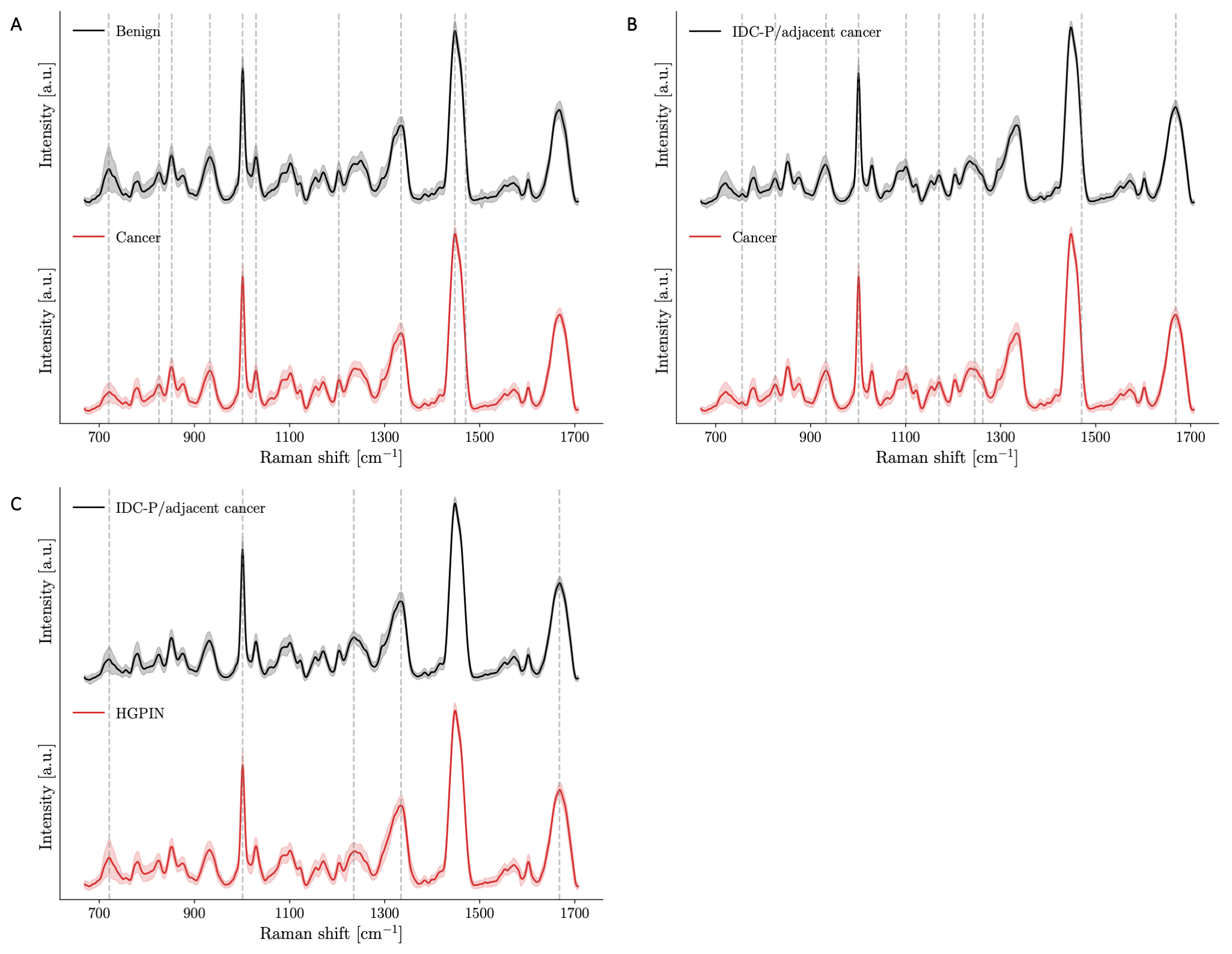

Supplement: S6 Fig — Average Raman spectra of benign prostatic glands and PC (A), IDC-P with adjacent cancer and PC (B), and IDC-P with adjacent cancer and HGPIN (C) from the CHUM cohort. Average spectra are shown (bold) with their associated variance (shaded area). Raman peaks (i.e., biochemical constituents of the tissue) that were dominant contributors to the classification were identified through a linear SVM with L1 regularization and are shown with dotted gray lines. (TIF) [file pmed.1003281.s006.tif]

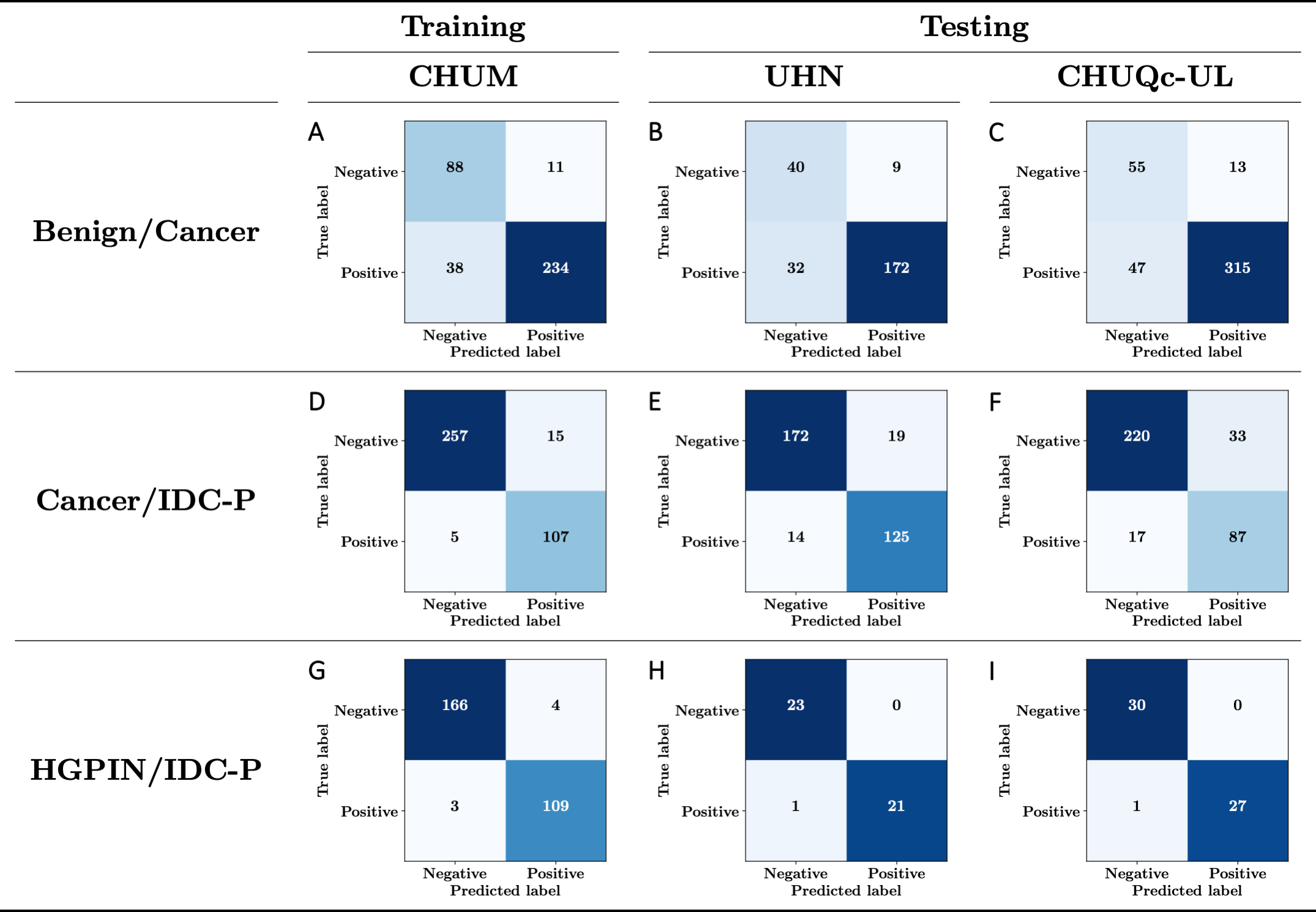

Supplement: S7 Fig — Confusion matrices associated with models differentiating between benign tissue, PC, IDC-P, and HGPIN in training and testing cohorts. In each panel (A–I), columns represent the predicted numbers for a given class while rows represent the numbers belonging to their true class (pathological labels). These numbers allow extraction of true positive, true negative, false positive, and false negative rates for each model in both training and testing sets. Numbers in each cell represent the number of cores, except for IDC-P in (D–G) and HGPIN in (G), which correspond to the total number of spectra. (TIF) [file pmed.1003281.s007.tif]
