# Supplementary material for: Identification of intraductal carcinoma of the prostate on tissue specimens using Raman micro-spectroscopy: A diagnostic accuracy case–control study with multicohort validation
Source: PLoS Med. 2020 Aug 14;17(8):e1003281. doi: 10.1371/journal.pmed.1003281 (PMC7428053; doi:10.1371/journal.pmed.1003281)
Supplement: S2 Table — (DOCX) [file pmed.1003281.s009.docx]

**S2 Table.** Classification performance when distinguishing lymphocyte clusters from prostate cancer with trained and tested independent cohorts.

| **Classification performance (%)** |  | **Training** |  | **Testing** | |
| --- | --- | --- | --- | --- | --- |
|  |  | **CHUM** |  | **UHN** | **CHUQc-UL** |
| Lymphocytes/Cancer |  |  |  |  |  |
| Sensitivity |  | 98 |  | 95 | 91 |
| Specificity |  | 99 |  | 96 | 93 |
| Accuracy |  | 98 |  | 95 | 93 |

The classification was performed using Support Vector Machine and L1-LinearSVC feature selection. (CHUM: Centre hospitalier de l’Université de Montréal; UHN: University Health Network; CHUQc-UL: Centre hospitalier universitaire de Québec-Université Laval).
