# Supplementary material for: Identification of intraductal carcinoma of the prostate on tissue specimens using Raman micro-spectroscopy: A diagnostic accuracy case–control study with multicohort validation
Source: PLoS Med. 2020 Aug 14;17(8):e1003281. doi: 10.1371/journal.pmed.1003281 (PMC7428053; doi:10.1371/journal.pmed.1003281)
Supplement: S3 Table — (DOCX) [file pmed.1003281.s010.docx]

**S3 Table.** Most important features used for the classification of lymphocytes and cancer within prostate tissue and their associated Raman peaks.

| **Feature**  **(cm^-1^)** | **Peak center**  **(cm^-1^)** | **Increased in** | **Main vibrational modes** | **Main molecules** |
| --- | --- | --- | --- | --- |
| 737 | 725-726 | Lymphocytes | C-S stretch, CH_2_ rocking | DNA/RNA (adenine), Protein |
| 787 | 781-786 | Lymphocytes | O-P-O | DNA/RNA (cytosine, uracil, thymine) |
| 1001/  1002/  1003 | 1000-1003 | Cancer | symmetric ring breathing | Protein (phenylalanine) |
| 1208/  1211 | 1206-1207 | Cancer | C-C_6_H_5_ stretch | Protein (phenylalanine, tryptophan, tyrosine) |
| 1223 | 1242-1250 | Cancer | Amide III | Protein (β-sheet) |
| 1300 | 1296-1305 | Lymphocytes | Fatty acid | Lipid |
| 1343/  1344 | 1338 | Cancer | CH_3_CH_2_ | DNA/RNA (adenine), collagen |
|  |  |  |  |  |
| 1431/  1451/  1452/  1453/  1454/  1455/  1456 | 1447-1450 | Lymphocytes | CH_2_ deformation | DNA/RNA, Protein, Lipid |

The feature selection algorithm used was a linear SVM with L1 regularization. Tentative molecular assignment of prostate Raman peaks based on literature findings [24-28, 38, 39].
